# Learning Curve for Using Intraoperative Neural Monitoring Technology of Thyroid Cancer

**DOI:** 10.1155/2019/8904736

**Published:** 2019-02-11

**Authors:** Ning Zhao, Zhigang Bai, Changsheng Teng, Zhongtao Zhang

**Affiliations:** Department of General Surgery, Beijing Friendship Hospital, Capital Medical University, Beijing Key Laboratory of Cancer Invasion and Metastasis Research, National Clinical Research Center for Digestive Diseases, Beijing 100050, China

## Abstract

We investigated the learning curve for using intraoperative neural monitoring technology in thyroid cancer, with a view to reducing recurrent laryngeal nerve injury complications. Radical or combined radical surgery for thyroid cancer was performed in 82 patients with thyroid cancer and 147 recurrent laryngeal nerves were dissected. Intraoperative neural monitoring technology was applied and the “four-step method” used to monitor recurrent laryngeal nerve function. When the intraoperative signal was attenuated by more than 50%, recurrent laryngeal nerve injury was diagnosed, and the point and causes of injury were determined. The time required to identify the recurrent laryngeal nerve was 0.5–2 min and the injury rate was 2.7%; injuries were diagnosed intraoperatively. Injury most commonly occurred at or close to the point of entry of the nerve into the larynx and was caused by stretching, tumor adhesion, heat, and clamping. The groups are divided in chronological order; a learning curve for using intraoperative neural monitoring technology in thyroid cancer surgery was generated based on the time to identify the recurrent laryngeal nerve and the number of cases with nerve injury. The time to identify the recurrent laryngeal nerve and the number of injury cases decreased markedly with increasing patient numbers. There is a clear learning curve in applying intraoperative neural monitoring technology to thyroid cancer surgery; appropriate use of such technology aids in the protection of the recurrent laryngeal nerve.

## 1. Introduction

With the rise in the incidence of thyroid cancer, increasing numbers of patients are undergoing total thyroidectomy, with consequential voice changes due to recurrent laryngeal nerve (RLN) injury, which has a serious impact on the patient's quality of life. In general, the reported RLN injury rate ranges from 1% to 13.3%. According to the Diagnosis and Treatment Guidelines for Patients with Thyroid Nodule and Differentiated Thyroid Cancer in China, the effective protection of the RLN is one of the key points in thyroid cancer surgery. In these guidelines, electrophysiological intraoperative neural monitoring (IONM) for RLN protection emerged after the regional protection method and fine dissection method. IONM has been developed over the past 30 years, and IONM has been clinically applied for nearly 20 years. Currently, IONM is considered required technology for thyroid operation in Europe and the USA and is rapidly being incorporated in China. Use of IONM is cost-effective for patients undergoing bilateral thyroid surgery [1]. A neural-monitored, staged surgical approach has been conducted and has demonstrated few significant adverse events in a small sample and represents an effective alternative to simultaneous bilateral surgery for the management of thyroid cancer with extensive neck metastases [2]. IONM involves applying a stimulating current to contract the dominant muscles through nerve conduction, and transmitting the electromyographic signals back to a nerve monitor through a signal reception electrode in contact with these muscles. The monitor analyzes the absence/presence and strength of the electromyographic signal to determine the nerve positioning and judge its function.

We have started using IONM (via direct RLN stimulation) during thyroid cancer surgery in July 2012 and have monitored its use by surgeons up to July 2014, by which time the General Surgery Department has completed 82 thyroid cancer operations. We used this information to assess the learning curve for use of IONM in this context.

## 2. Methods

### 2.1. General Information

All cases included in this study were compatible with the International Standards Guidelines for Electrophysiological Recurrent Laryngeal Nerve Monitoring during Thyroid and Parathyroid Surgery [3]. The motion of the bilateral vocal cords was inspected by using a fiberoptic laryngoscope either preoperatively or on the first day after the operation. According to the thyroid cancer TNM staging criteria (7^th^ edition, 2010) prepared by the AJCC, all patients were in TNM stage I–III. All patients signed informed consent forms and this study was approved by the ethics committee of our hospital.

### 2.2. IONM Method

Combined inhalation and intravenous anesthesia were adopted for thyroid cancer surgery. Short-acting muscle relaxant was used for intubation. No muscle relaxant was added during the operation. The NIM-Response 3.0 intraoperative nerve monitoring system (Medtronic; Dublin, Ireland) and laryngeal electromyography (EMG)-enhanced tracheal intubation tubes were used. During intubation, the blue zone was placed outside of the glottis to ensure that the two groups of electrodes leads on the intubation tubes were placed in contact with the vocal cords on both sides. The current intensity used for the stimulating electrode during surgery was 1.0–3.0 mA. The method for seeking the RLN is termed the “four-step method” [3]: first, the two layers of the thyroid capsule were separated outward, until the carotid sheath was exposed; next, the vagus nerve was sought between the common carotid artery and the internal carotid artery; then, the probe was used to stimulate the vagus nerve, and the maximum amplitude was recorded on electromyography as the V1 signal. The posterior part of the thyroid capsule was then carefully dissected, the probe used to detect the position of the RLN near the trachea esophagus groove, and the maximum amplitude recorded as the V1 signal and R1 signal. The time between obtaining the V1 signal and obtaining the R1 signal represented the time required to identify the RLN (i.e., the time from the point of incising the neck, revealing the thyroid surgical capsule, to finding the recurrent laryngeal nerve, revealing 1 cm of the nerve in order to confirm it as the RLN based on appearance). The isthmus of the thyroid gland was then resected, the pretracheal fascia was separated, the inferior and superior thyroid arteries were ligated, the thyroid lobe was removed from the tracheal side, and the V2 and R2 signals were then obtained after clearing away lymph glands in zone VI.

### 2.3. Diagnosis of RLN Injury

The signal attenuation range was determined: if the degree to which the R2 signal was weaker than the R1 signal exceeded 50% [4], RLN injury was diagnosed, except in cases of movement of the tracheal tube, improper use of muscle relaxants, or equipment connection and instrument failure. The point of injury was judged as follows: if the R2 signal was lower than the R1 signal closer to the larynx, and the R2 signal was equal to the R1 signal further away from the larynx, after careful monitoring by means of the probe along the RLN, this point is injury point. If the R2 signal was smaller than the R1 signal along the length of the RLN, the injury point was considered to be within the larynx. The causes of RLN injury were established after determining the injury point and included injury due to heat, stretching, and clamping.

### 2.4. Treatment for RLN Injury

If RLN injury was identified, 40 mg of methylprednisolone was immediately injected intravenously, during the operation, and ribbon gauzes soaked in dexamethasone were applied to the surface of the RLN for more than 10 min; they were then removed before suturing the incision. After the operation, 40 mg of methylprednisolone was administered once daily, via intravenous injection, for 2 successive days, and 0.5 mg mecobalamin tablets were administered orally twice a day until RLN function had recovered. If the patient's voice was restored to normal and the closure of the vocal cords was considered normal upon fiberoptic laryngoscope examination, the patient's RLN function was considered to have recovered completely.

### 2.5. Statistical Analysis

Statistical software SPSS19.0 (SPSS, Inc., Chicago, IL, USA) was used to conduct the statistical analysis. The measurement data that were demonstrated as the mean ± standard deviation (SD), such as Baseline characteristics. One-way analysis of variance was used for time to identify among groups the recurrent laryngeal nerve by SPSS 19.0. Statistical significance was set at a P value of <0.05.

## 3. Results

**Figure 1:**
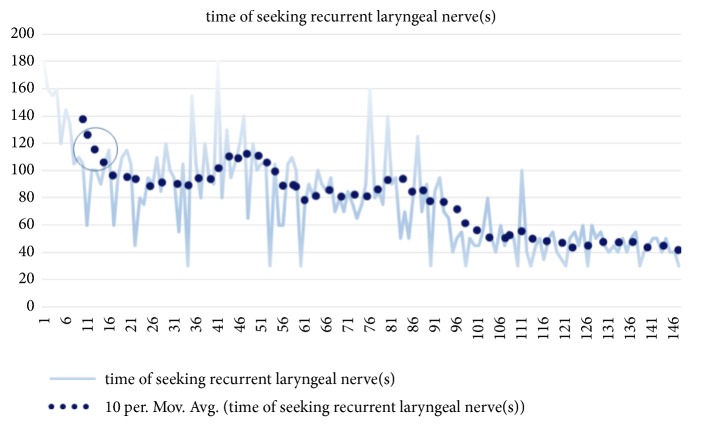
Mean operation time for all cases. The operative time decreased markedly after the first 10 cases.

The details of the consecutive 82 cases are shown in Table 1. The same team of doctors performed 61 radical excisions and 21 combined radical operations, among which 8 cases were reoperation cases. A total of 147 thyroid gland lobes were removed. The time spent in seeking the RLN in the excision of 147 thyroid gland lobes ranged from 0.5 to 2 minutes. As shown in Figure 1, the time required to identify the RLN decreased gradually as the number of cases increased.

Four patients were diagnosed with RLN injury intraoperatively (injury rate of 2.7%; 4/147). Fiberoptic laryngoscopy postsurgically indicated that the patients' vocal cord motion had weakened or remained stable during 12 months. The signal attenuation range, injury point, injury cause, and RLN function recovery time after treatment are shown in Table 2. The RLN signal showed no obvious attenuation or showed an attenuation range of less than or equal to 10%, during the excision of 143 thyroid gland lobes. Postsurgical fiberoptic laryngoscopy revealed that these patients' vocal cord motion was normal.

Groups were established based on chronological time, and each group consisted of surgery of 30 thyroid gland lobes. The time to identify the RLN during surgery and the number of injury cases (Table 3) were recorded, and the time for identifying the RLN was compared using one-way analysis of variance (Table 4). There was no statistically significant difference among the groups in terms of the number of RLN injury cases (p = 0.486; Fisher's exact test).

## 4. Discussion

IONM was used for continuous monitoring during 82 thyroid cancer operations during which 147 thyroid lobes were removed. The time for identifying the RLN by means of IONM was 0.5–2 min, based on the fine dissection method. Among the 82 patients, 4 patients suffered RLN injury (injury rate: 2.7%), which was identified intraoperatively by IONM. The injury point was located near the larynx and the injury causes included stretching, tumor adhesion, heat, and clamping. All injuries were treated timely.

The reported complications of RLN injuries may be related to positioning of electrodes for IONM at the larynx, obstructing the endotracheal tube; the drugs used for anesthesia; and the effects of electrical stimulation at nerve structural and systemic levels [5]. Stretch injury to the RLN is the most common type of RLN injury. The thyroid capsule tissue near the point of RLN entry into the larynx compresses the RLN to the tracheal surface; thus, RLN injury can occur when separating the lobe and picking it up towards the inside, particularly when the malignant tumor is stuck to the thyroid capsule and is located close to the RLN, even when the mass is not particularly large. Heat injury is another common type of RLN injury. High temperature conduction causes albuminous degeneration of the RLN when using electrical equipment, such as an electrotome, bipolar electrocoagulator, ultrasound knife, and energy platform, resulting in heat injury. Clamping injury is generally caused when the RLN is accidentally clamped when attempting to stop hemorrhage of the posterior membrane.

Although RLN can be found relatively rapidly by using IONM intraoperatively, it has to be dissected after determining its position, to complete the exposure. This dissection process is very likely to cause injury. Using IONM can help to detect whether RLN function has been affected after performing separation and hemostasis.

In our study, the groups are divided in chronological order, with each group consisting of 30 RLNs; the time of seeking RLN and the number of injury case number have dropped obviously in the latter group. In addition, all injury cases occurred in the top 3 groups, which indicated that the use of IONM is beneficial to protection of the RLN. Understanding the learning curve for applying IONM technology may facilitate the use of IONM, which can assist in locating the RLN prior to exposing it via dissection, and it will be faster than locating it visually. More importantly, although macroscopic RLN injury, i.e., its complete disruption or mechanical damage, can be observed intraoperatively without IONM; in non-macroscopic RLN injury, the nerve appears to be in good condition, but is functionally damaged, and the existence, location, and cause of such injury can be difficult to judge without IONM. Without such knowledge, another such injury is likely to occur in subsequent operations. Using IONM, surgeon can determine the cause of injury and can try to avoid recurrence. The injury causes in the 4 cases in this study could be clearly determined, allowing surgeons to adjust their surgical technique, and, subsequently, no similar injuries occurred. With accumulation of operative cases, the RLN injury cases declined in this study.

It has been disputed whether using IONM can reduce RLN injury rate. Ide et al. [6] compared 104 cases with IONM and 100 cases without IONM and found RLN injury rates of 6.8% and 7.5%, respectively; this difference was not statistically significant. Shindo and Chheda [7] have reported a study of 684 cases, in which they dissected 1043 RLNs; the unilateral RLN injury rates in the IONM group and control group were 2.09% and 2.96%, respectively, which were not statistically significantly different. Atallah et al. [8] have reported their experience of using IONM in “high-risk” thyroid operations; compared with a control group, the temporary RLN injury rates were 8.8% and 9.1%, respectively, while the permanent RLN injury rates were 3.9% and 3.8%, respectively, again without statistical significance. Dralle [9] retrospectively analyzed 16488 thyroid operations in a multicenter study; the study included 29998 RLNs in total. The RLNs were divided into 3 groups: in the first group, the nerve was not exposed; in the second group, the RLN was exposed under direct vision; and in the third group, the RLN was exposed under direct vision by using IONM. They found that the RLN injury rate was not reduced in the third group as compared with the second group. Therefore, it seems that using IONM does not reduce the RLN injury rate.

The International Neural Monitoring Study Group has issued specific operation instructions [10], including the four-step monitoring method and vocal cord motion laryngoscopy (direct laryngoscopy or fiberoptic laryngoscopy) before and after surgery. The type of IONM we used was direct RLN stimulation. Chiang et al. [11] have reported that the RLN injury rate declined to 0.8%, from 6.4%, after confirming the tracheal intubation position again during surgery, according to standard operation procedures. Barczynski et al. [12] reported randomized control trial research results and stated that using IONM can reduce the temporary RLN injury rate, particularly among patients in a high-risk group (malignant thyroid tumor, hyperthyroidism, goiter behind the sternum, huge goiter). Therefore, IONM can protect the RLN in difficult thyroid operations. In 2013, the Clinical Guidelines for Intraoperative Nerve Electrophysiology Monitoring in Thyroid and Parathyroid Operations (Chinese version) [3] stated the indications for IONM use. According to our experience, the use of IONM should be considered under the following circumstances: (1) patients with malignant thyroid tumor who require lobe excision and central compartment lymph node dissection; (2) patients with thyroid reoperation; (3) patients with huge goiter and goiter behind the sternum; (4) patients with thyroid tumors in the posterior region of the gland; (5) patients with situs inversus or “arteria subclavia” and nonrecurrent laryngeal nerve identified before the operation; and (6) if patients require surgery for unilateral vocal cord paralysis and contralateral thyroid gland lobe removal.

Although the data presented in this study are not sufficient to conclude the impact of using IONM on RLN injury rate, it does indicate that there is a learning curve in use of IONM technology. According to our experience, the clinical application of IONM has the following significance: (1) it shortens the time for identifying the RLN; (2) it reduces RLN injury and predicts nerve function after surgery; (3) it helps to determine the RLN injury point and causes; (4) it assists in timeously identifying nerve injury on one side and judging whether the operation should be delayed for the other side; and (5) it helps to identify whether any voice change after surgery was caused by RLN injury.

Nevertheless, use of IONM has some limitations: (1) RLN injury cannot be completely avoided. With invasive neoplasm, adhesion or a large mass, and anatomic variation, such injury will occur during the process of tumor separation, even though the nerve position can be detected [13]. (2) The surgeon should have the capacity to judge and avoid common mistakes by professional training, including training in equipment connection, positioning of tracheal intubation, and understanding the impact of muscle relaxants. (3) IONM cannot replace good surgical technique and IONM cannot be used to protect the nerve in the absence of dissection experience [14]. (4) In surgery under local anesthesia, the nonspontaneous contraction of the laryngeal muscle will generate marked interference signals, because the patient is awake. At present, no equipment can resolve this issue and, thus, application of IONM in this context is limited. (5) IONM involves increased costs, which are not covered by health insurance, limiting its application due to economic factors.

In conclusion, there is a technology learning curve in the application of IONM in thyroid cancer surgery, but the use of IONM may assist in protecting RLN during fine dissection.

## Figures and Tables

**Table 1:** Characteristics of the patients.

Baseline characteristics	Number/mean ± SD
Age (years)	46.6 ± 12.7
Sex ratio (F:M)	2.9:1
Radical surgery (n): combined radical surgery (n)	61:21
Tumor range (unilateral: bilateral) (n)	62:20
Tumor size (cm)	1.1 ± 0.8
Tumor number (n)	1.4 ± 0.8
Retrieved cervical central lymph nodes (n)	5.5 ± 3.8
Metastatic cervical central lymph nodes (n)	1.7 ± 1.5
Dissected recurrent laryngeal nerve (n)	147
pathological pattern (papillary/medullary) (n)	81/1

F: female; M: male.

**Table 2:** Case statistics of RLN injury.

Case	Lobe	Signal attenuation	Injury point	Injury cause	Recovery (months)
1	Left	70%	The point of entry into the larynx	Excessive stretching of RLN when picking up thyroid lobes	0.5
2	Left	100%	1 cm from the point of entry into the larynx	Tumor was adhered to RLN.	No recovery in 12 months.
3	Left	90%	The point of entry into the larynx	Heat injury caused by ultrasound knife during separating.	6
4	Right	90%	1 cm from the point of entry into the larynx	Clamping of vessel to stop hemorrhage included and injured the RLN	3

RLN: recurrent laryngeal nerve.

**Table 3:** Distribution of time to identify the RLN and injury across groups.

Group	Case	Time of seeking RLN (s)	Number of cases with injury
1	30	105 ± 34	2
2	30	100 ± 31	1
3	30	85 ± 24	1
4	30	50 ± 15	0
5	27	45 ± 9	0

Total	147	78 ± 35	4

RLN: recurrent laryngeal nerve.

**Table 4:** One-way analysis of variance for time to identify the recurrent laryngeal nerve among groups.

P value	Group 1	Group 2	Group 3	Group 4	Group 5
Group 1	/	0.436	0.002	0.000	0.000
Group 2	0.436	/	0.020	0.000	0.000
Group 3	0.002	0.020	/	0.000	0.000
Group 4	0.000	0.000	0.000	/	0.448
Group 5	0.000	0.000	0.000	0.448	/

## Data Availability

The data used to support the findings of this study are available from the corresponding author upon request.
